# Circular RNA CDR1as sponges miR-7-5p to enhance E2F3 stability and promote the growth of nasopharyngeal carcinoma

**DOI:** 10.1186/s12935-019-0959-y

**Published:** 2019-10-01

**Authors:** Qiong Zhong, Juncong Huang, Jiawang Wei, Renrui Wu

**Affiliations:** Department of Oncology, People’s Hospital of Ganzhou, No. 18 Mei Guang Avenue, Ganzhou, 341000 Guangdong People’s Republic of China

**Keywords:** NPC, Glucose metabolism, CircRNA CDR1as, miR-7-5p, E2F3

## Abstract

**Background:**

Circular RNA (circRNA) CDR1as plays an important role in the occurrence and development of human tumors. The purpose of this study is to investigate the molecular mechanism of circRNA CDR1as in the development of nasopharyngeal carcinoma (NPC).

**Methods:**

The mRNA expressions of circRNA CDR1as, miR-7-5p, and E2F3 were detected by qRT-PCR. The effects of circRNA CDR1as, miR-7-5p, and E2F3 on NPC cells were investigated using cell counting kit-8 (CCK8) method, colony formation assay, and representative metabolite assay. The molecular mechanism of circRNA CDR1 in NPC was studied by bioinformatics and luciferase reporter assay. In addition, the biological activity of circRNA CDR1as was also investigated in NPC xenograft tumor mice model.

**Results:**

The results showed that the circRNA CDR1as expression was significantly up-regulated in NPC tissues by comparison with non-tumor NPE tissues (*p *< 0.01), suggesting that circRNA CDR1as was associated with poor prognosis in NPC patients. Moreover, circRNA CDR1as could up-regulate E2F3 expression by binding miR-7-5p, and promote the growth and glucose metabolism of NPC cells. Meanwhile, circRNA CDR1as could promote NPC progression through the negative regulation of miR-7-5p in the xenograft tumor model.

**Conclusion:**

CircRNA CDR1as promoted the occurrence and development of NPCs by successively up-regulating the expression of miR-7-5p and E2F3, suggesting CircRNA CDR1as as a potential target for the treatment of NPC patients.

*Trial registration* The study was approved by the cancer center’s institutional research ethics committee on Oct 18, 2008 (2008GZ2847462)

## Background

Nasopharyngeal carcinoma (NPC) is one of the most common head and neck malignancies, which can be easily induced by environmental factors, genetic factors, and EB virus infection [1, 2]. NPCs have the pathological characteristics of high incidence, rapid deterioration, and poor prognosis and most NPC patients have developed to the middle- and late-stage by the time of diagnosis [3, 4]. Radiotherapy is dominated in the treatment of NPC due to the high sensitivity, but NPC cells are prone to distant metastasis and recurrence after treatment [5]. Therefore, gene therapy for NPC becomes popular. Glucose is the primary energy source of cells in the body. Normal cells generate ATP through oxidative phosphorylation under aerobic conditions and glycolysis under anaerobic conditions [6, 7]. The metabolism of malignant tumor cells is different from that of normal cells, and one of the main characteristics is the increase of glucose metabolism [8]. Even under aerobic conditions, most tumor cells metabolize glucose into lactic acid through glycolysis to produce ATP, which is called aerobic glycolysis or Warburg effect [9]. Therefore, the increase of glucose metabolism provides favorable conditions for the growth, proliferation, metastasis, and invasion of tumor cells.

CircRNAs are endogenous non-coding RNAs with functions similar to long non-coding RNAs [9, 10]. As competitive endogenous RNAs (ceRNA), CircRNAs can bind to miRNAs and inhibit their functions [11]. Cerebellar degeneration associated protein 1 antisense transcript (CDR1as) is the circular natural antisense transcript (NAT) of the CDR1 gene, which binds to miR-7 and acts as a molecular sponge of circRNA [12]. The precursor sources of miR-7 include miR-7-1, miR-7-2, and miR-7-3, among of which miR-7-5p is the mature of miR-7 and closely related to the occurrence and development of tumors [13]. Recent studies have found that miR-7-5p is an endogenous tumor suppressor gene that can target and inhibit lymphoma, gastric cancer, glioma, and other malignant tumors [14, 15]. CircRNA CDR1as contains 70 binding sites of miR-7 and can inhibit the function of miR-7 and indirectly promote the stability of miR-7 target genes [16–19].

E2F transcription factor 3 (E2F3) belongs to the member of E2F transcription factor family and is involved in the regulation of cell proliferation and cell cycle [20]. It has found that interference of E2F3 gene expression could prevent the cell cycle of bladder cancer 5637 cells in G1 phase and promote apoptosis [21]. However, the mechanism of circRNA CDR1as, miR-7-5p, and E2F3 in NPC is still unclear. In the present study, the specific molecular mechanism of circRNA CDR1as in NPC tissues and cells was explored by qRT-PCR, CCK8, colony formation test, representative metabolite analysis, luciferase reporter analysis and NPC xenograft tumor model. It may provide a certain theory for the targeted treatment of NPC.

## Materials and methods

### Materials

#### Organizational collection

Non-tumor NPE biopsy samples (n = 20) and NPC biopsy samples (n = 44) from People’s Hospital of Ganzhou were selected for the determination of CDR1as gene expression. NPC patients diagnosed by histopathological examination from 2009 to 2012 were selected. NPC tissue samples obtained by biopsy were collected. All the samples were immersed in the RNA-Later reagent at 4 °C for overnight, and then deposited at − 80 °C. Clinical characteristics of all patients were collected from the medical records (Table 1). The study was approved by the cancer center’s institutional research ethics committee.

**Table 1:** Correlation between CDR1as expression and clinical pathological characteristic in NPC. Using the median CDR1as value as cutoff

Parameters	Group	n	CDR1as expression		*p* value
			High (n = 22)	Low (n = 22)	
Age (years)	≤ 60	16	7	9	0.322
	> 60	28	15	13	
Gender	Female	12	5	7	0.442
	Male	32	17	15	
T classification	T1–T2	25	13	10	0.168
	T3–T4	19	14	11	
N classification	N0–N1	18	10	8	0.195
	N2–N3	26	12	14	
Clinical stage	I–II	11	3	8	0.002**
	III–IV	33	19	14	
Distant metastasis	Yes	15	7	8	0.622
	No	29	15	14	
Loco-regional recurrence	Yes	13	4	9	0.037
	No	31	18	13	

n = 44, ** *p* < 0.01

#### Cells

Human nasopharyngeal epithelial cells NP69 and N5-Tert were cultured in keratinocyte serum-free medium (Invitrogen, Carlsbad, CA, USA). NPC cell lines (CNE1, SUNE1, 6-10b, 5-8f, HNE1, HK1 and HONE1) were cultured using RPMI 1640 medium (Gibco, 11875093, Fisher Scientific, USA) containing 5% FBS (Hyclone, SH3007103, Fisher Scientific, USA). All cells were incubated at 37 °C in a humidified atmosphere with 95% air and 5% CO_2_.

### Quantitative real-time PCR (qRT-PCR)

The tissue samples were ground into powder in a liquid nitrogen precooled mortar. Total RNA was extracted from tissues and cells using Trizol reagent (Invitrogen, 15596026, Fisher Scientific, USA), and the specific steps were strictly carried out according to the instructions of the kit. Then, the Thermo Script RT-PCR system (Invitrogen, 11731015, Fisher Scientific, USA) was used to reverse transcribe 1 μg total RNA into cDNA. qRT-PCR was performed using Light Cycler 480 SYRB Green I Master Mix (04707516001, Roche, Indianapolis, USA). GAPDH was regarded as an internal reference. We used the 2^−ΔΔCt^ method to calculate the relative expression level. All experiments were carried out in three parallel experiments. The primer sequences used in the experiment were shown in Table 2.

**Table 2:** The sequences of specific primers

Gene name	Primer sequence (5′ to 3′)
CDR1as	Forward: 5′-CCAATTGCGCCTTCAGGCTA -3′ Reverse: 5′-CGGGGGAGTCCTTACCCACA-3′
miR-7-5p	Forward: 5′-CAGGGAGGCGTGGATCACTG-3′ Reverse: 5′-CGTCG GGGGCTCATGGAGCGG-3′
E2F3	Forward: 5′-CTTGGGAGAGTTGCTTCGAAA-3′ Reverse: 5′-GCGCTGTGCATCGCAG-3′
GAPDH	Forward: 5′-CGCGAGAAGATGACCCAGAT-3′ Reverse: 5′-GGGCATACCCCTCGTAGATG-3′

### Determination of colony formation

Specific siRNA transfected cells (400 cells/well) were inoculated in 6-well plates and cultured for 14 days. After culture, PBS was used for washing, and methanol was used for fixation for 15 min. Next, the samples were stained at room temperature with 0.5% crystal violet in 20% methanol solution for 15 min. After washing, 6-well plates were photographed, and the colony formation was analyzed. All experiments were carried out in three parallel experiments.

### Oligonucleotides

Oligonucleotides, miR-7-5p inhibitor, the siRNA of miRNA and E2F3 were purchased from Gene Pharma (Shanghai, China). Oligonucleotides were transfected into cells using Lipofectamine 2000 reagent (11668027, Invitrogen, Fisher Scientific, USA). Oligonucleotide sequences were shown in Table 3.

**Table 3:** Sequences of oligonucleotides

Gene name	Primer sequence (5′ to 3′)
miR-7-5p mimic	5′-AAAAGUGCUUACAGUGCAGGUAG-3′
si-CDR1as 1	5′-CCCACAACAUGAAAGAAACTT-3′
si-CDR1as 2	5′-GCUAGACCUUTGGAACCAGAT-3′
negative control	5′-UUCUCCGAACGUGUCACGUTT-3′

### CCK-8 assay

The proliferation ability of NPC cells was determined by CCK-8 kit (C0037, Beyotime, China) assay. The transfected NPC cells (5 × 10^3^ cells/well) were inoculated in the 96-well culture plate. The culture medium was replaced with fresh medium containing 10% CCK8 after cell culture for 12, 24, 36, and 48 h, respectively. After incubation for 3 h, the absorbance value was determined using a microplate reader at 450 nm.

### Representative metabolite assay

The contents of glucose and lactic acid in cells were determined using the glucose and lactic acid assay kit (K606, K638, Biovision, USA), and the specific operation steps were strictly in accordance with the instructions of the kit. The absorbance value at 570 nm was determined by a microplate reader. Glucose and lactate concentrations were calculated according to the standard curve. To remove as much of the effect as possible, the cells were measured and counted for the next 24 h. Pico Probe Lactate Fluorometric Assay Kit (K638, Biovision, USA) was used to determine lactic acid levels in mouse tumor tissues. The content of ATP in cells was determined using the ATP kit (Biovision, Milpitas, USA).

### Western blot

Total protein in cells was extracted with RIPA buffer (KenGEN, China) and quantified with BCA protein concentration kit (P0012S, Beyotime, China). The extracted protein was separated by 10% SDS-PAGE and transferred onto PVDF membrane (IPVH00010, Millipore, USA). After sealing with 5% skim milk for 2 h, the PVDF membrane was washed three times with PBST. Then, the PVDF membrane was incubated with E2F3 antibody (ab50917, 1:2000, Abcam) overnight and washed with PBST for three times. The second antibody was then incubated at room temperature for 1 h. GAPDH (ab9485, 1:2500, Abcam) was used as an internal reference.

### Luciferase reporter assay

E2F3 3′-UTR fragment containing specific miR-7-5p binding sequence was cloned and inserted into the pmir-report vector. CircRNA CDR1 fragments containing miR-7-5p binding sites were inserted into the pmir-report vector. MiR-7-5p mimics or related miRNA and recombinant plasmids or empty pmir-report vectors were co-transfected into NPC cells. Reporter Assay System (E1910, Promega, USA) was used to detect luciferase activity in cells.

### Xenograft tumor model experiment

18 nude mice were purchased from Beijing laboratory animal research center. Three mice were injected subcutaneously with HK1 cells. When the tumor diameter was 5 mm, the tumor was removed and cut into 1–2 mm^3^ slices, which were further inoculated into the subcutaneous tissues of another 15 mice. The 15 mice were randomly divided into three groups: control group, circRNA CDR1as siRNA group, and miR-7-5p mimic group. Mice in each group were injected with the corresponding reagent every 3 days, and tumor volume was measured. Tumor tissue was removed and weighed after 30 days. The study was approved by the laboratory animal ethics committee of the People’s Hospital of Ganzhou. As described in the literature [22], the immunohistochemical staining was performed on the tumor tissue, and the tissue sections were co-incubated with E2F3 antibody (ab50917, 1:100, Abcam).

### Statistical analysis

SPSS 13.0 software was used to analyze the data in this study, and all data were expressed as mean ± SD. T-test or one-way variance was used for significance analysis. Pearson correlation analysis was performed using MATLAB, and Kaplan–Meier was used to analyze the survival rate. *p *< 0.05 was considered statistically significant. All experiments were three independent parallel experiments.

## Results

### Determination of CDR1as expression level in NPC tissue and cells

**Fig. 1:**
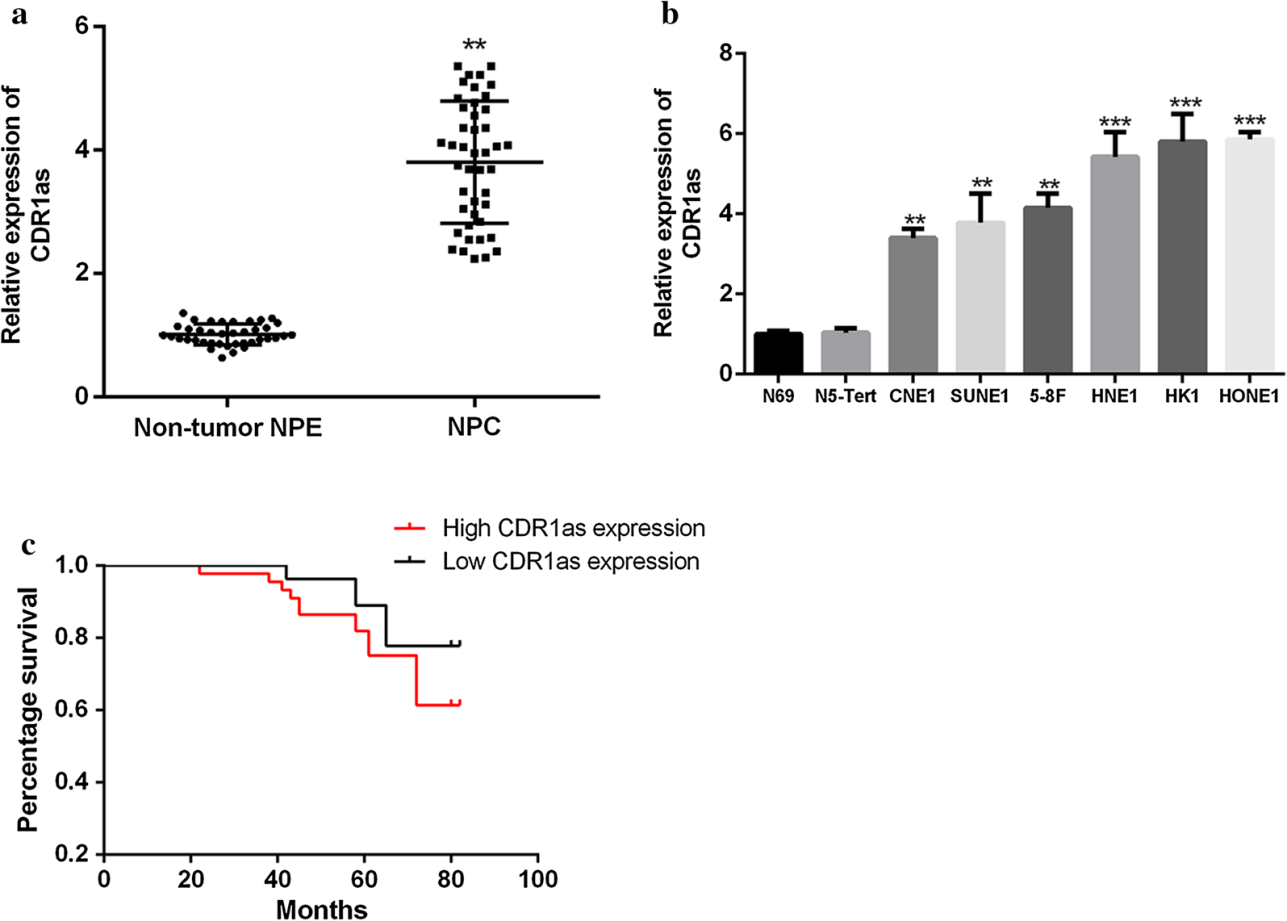
Detection of the mRNA expression of CDR1as in NPC tissues and cells by qRT-PCR. **a** The mRNA expression of CDR1as in NPC and NPE tissues. **b** The mRNA expression of CDR1as in NPC cell lines and NPEC. **c** The relationship between CDR1as expression and overall survival percentage by Kaplan–Meier. Using the median CDR1as value as cutoff. **p < 0.01, ***p < 0.001

In the present study, non-tumor NPE biopsy samples (n = 20) and NPC biopsy samples (n = 44) were selected for the determination of CDR1as mRNA expression in tissues. The results showed that the mRNA expression of CDR1as in NPC tissues was significantly increased, compared with non-tumor NPE tissues (Fig. 1a, *p* < 0.01). Meanwhile, NPEC (N69 and N5-Tert) was used as the control cells, and the expression of circRNA CDR1as in NPC cell lines (CNE1, SUNE1, 6-10B, 5-8F, HNE1, HK1, and HONE1) was determined by qRT-PCR. As shown in Fig. 1b, there was no significant difference in mRNA expression of CDR1as between N69 and N5-Tert cells (*p *> 0.01). Compared with N69 cells, the mRNA expression of CDR1as in NPC cell lines was significantly up-regulated (*p *< 0.01), and the mRNA expression of CDR1as in HK1 and HONE1 cells was the highest (*p *< 0.001). Therefore, HK1 and HONE1 cells were selected for subsequent experiments. In addition, Pearson correlation analysis showed that the high expression of circRNA CDR1as in the tissues of NPC patients was correlated with clinical staging (*p *< 0.01), but not related to other factors (Table 1). Kaplan–Meier analysis showed that the high expression of circRNA CDR1as was associated with high survival of NPC patients (Fig. 1c). The above results confirmed that circRNA CDR1as was highly expressed in NPC tissues and cell lines, which might be related to the promotion of NPC development.

### Effects of circRNA CDR1as on the growth and glucose metabolism of NPC cells

**Fig. 2:**
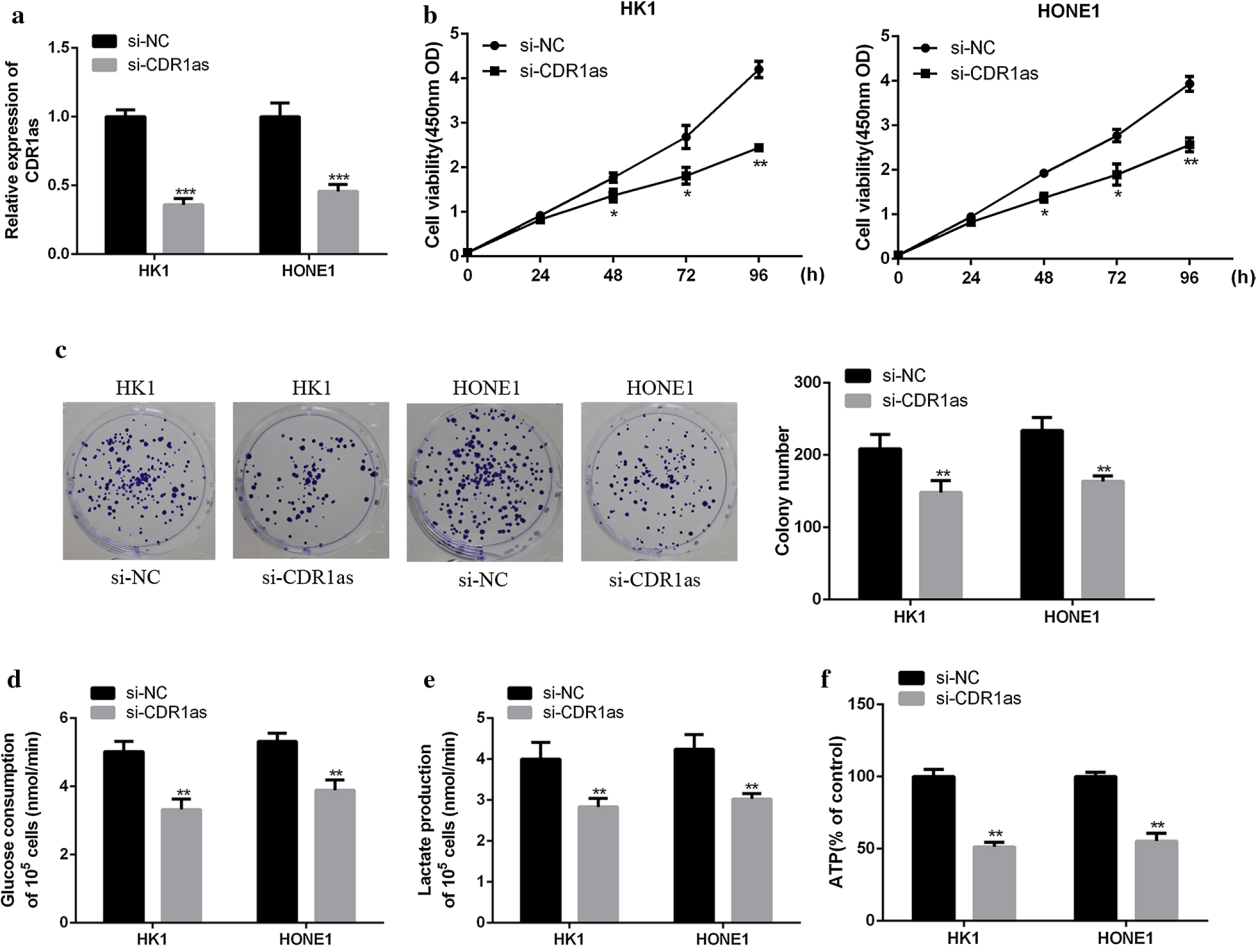
Effects of circRNA CDR1as on the growth and glucose metabolism of NPC cells. **a** qRT-PCR analysis on the transfection efficiency of si-CDR1as. **b** Viability determination of HK1 and HONE1 cells by CCK8. **c** Proliferation measurement of HK1 and HONE1 cells by colony formation. **d** Determination of glucose consumption in HK1 and HONE1 cells. **e** Determination of lactic acid production in HK1 and HONE1 cells. **f** Determination of ATP production in HK1 and HONE1 cells. *p < 0.05, **p < 0.01, ***p < 0.001

To determine the effect of circRNA CDR1as on NPC cells, si-CDR1as or si-NC were transfected into HK1 and HONE1 cells, respectively. As shown in Fig. 2a, circRNA CDR1as expression in HK1 and HONE1 cells transfected with si-CDR1as was significantly lower than that in the si-NC transfection group (*p *< 0.001), indicating successful transfection. In addition, CCK8 assays and colony formation assay showed that the interference of circRNA CDR1as expression significantly inhibited the viability and proliferation of HK1 and HONE1 cells (Fig. 2b, c). Meanwhile, Fig. 2d, e showed that the interference of circRNA CDR1as expression also significantly reduced glucose consumption (*p *< 0.01) and lactic acid content (*p *< 0.01) in HK1 and HONE1 cells. Furthermore, the interference of circRNA CDR1as expression significantly inhibited ATP production in HK1 and HONE1 cells (Fig. 2f, *p *< 0.01). These results indicated that circRNA CDR1as could promote the growth and glucose metabolism of NPC cells.

### Identification of miR-7-5p as the target gene of CDR1as in NPC

**Fig. 3:**
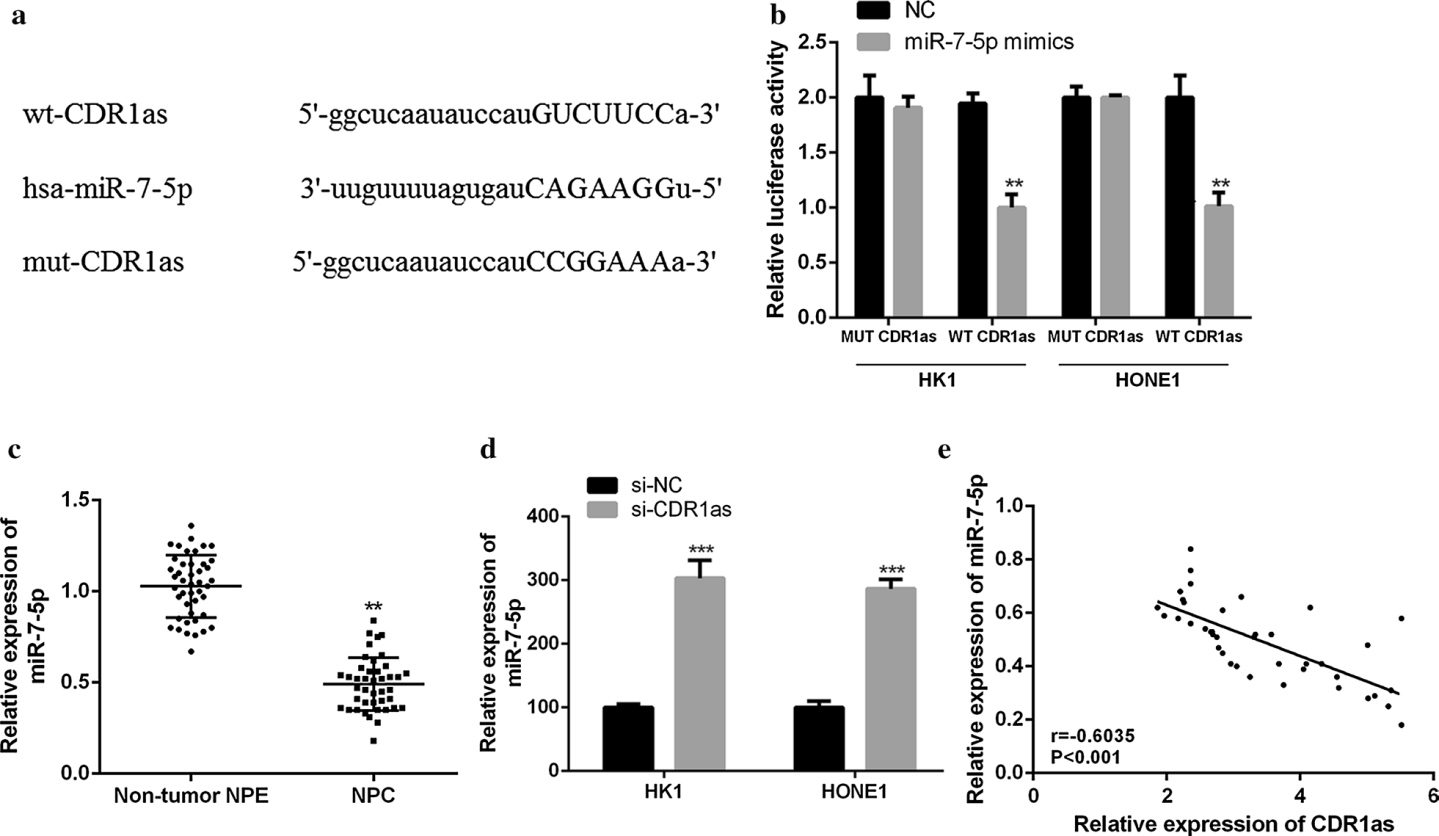
MiR-7-5p acted as the target gene of CDR1as in NPC. **a** Prediction of the binding sites between CDR1as and miR-7-5p by Starbase bioinformatics analysis. **b** The constructed WT-CDR1as or MUT-CDR1as and miR-7-5p mimics or control miRNA were co-transfected into HK1 and HONE1 cells to detect luciferase activity. **c** Detection of the miR-7-5p expression in tissues by qRT-PCR. **d** Detection of the miR-7-5p expression in HK1 and HONE1 cells by qRT-PCR. **e** Correlation analysis of CDR1as and miR-7-p in NPC tissues. **p < 0.01, ***p < 0.001

It has been reported that circRNA can be used as a competitive endogenous RNA (ceRNA) to bind functional miRNA and release miRNA-targeted mRNA during the occurrence and development of tumor, thus regulating gene expression [23]. In this experiment, bioinformatics predicted that miR-7-5p might be the target of CDR1as, and the binding site between CDR1as and miR-7-5p was shown in Fig. 3a. The constructed wild-type (WT) CDR1as or mutant-type (MUT) CDR1as were transfected into HK1 and HONE1 cells together with miR-7-5p mimic or control miRNA. Double luciferase reporter assays showed that the luciferase activity in HK1 and HONE1 cells co-transfected by WT CDR1as and miR-7-5p mimics was significantly lower than that in the control group (Fig. 3b, *p *< 0.01). In addition, the miR-7-5p expression in NPC tissues was significantly down-regulated, compared with non-tumor NPE tissues (Fig. 3c, *p *< 0.01). Importantly, the interference of CDR1as expression significantly promoted miR-7-5p expression in HK1 and HONE1 cells (Fig. 3d, *p *< 0.001). Moreover, the correlation analysis showed that there was a significantly negative correlation between the expression of CDR1as and miR-7-5p in NPC tissues (Fig. 3e, *p *< 0.001). The above results confirmed that miR-7-5p was the target gene of CDR1as in NPC.

### Identification of E2F3 as the target gene of miR-7-5p in NPC cells

**Fig. 4:**
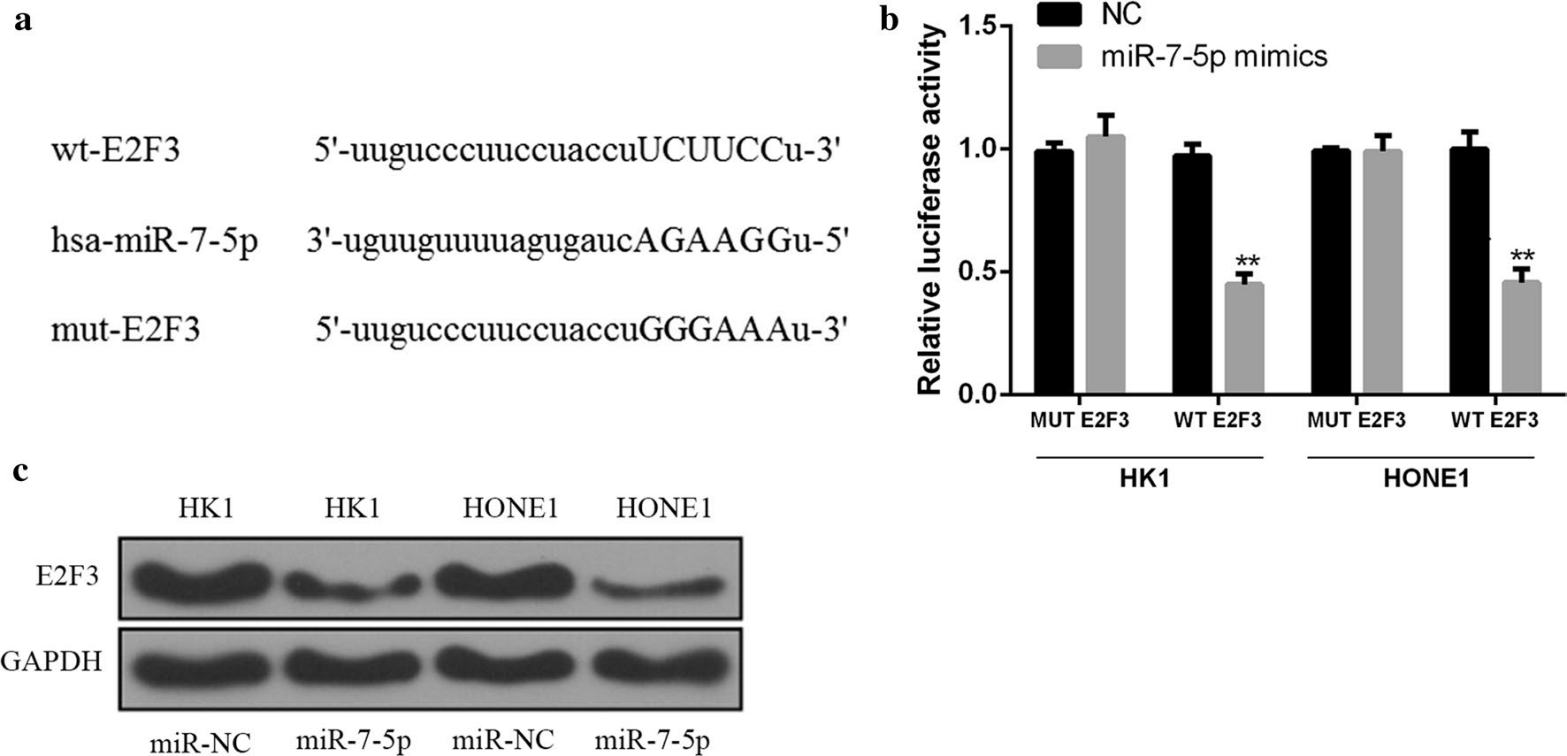
E2F3 acted as the target gene of miR-7-5p in NPC cells. **a** Prediction of the binding sites of miR-7-5p in E2F3 3′-UTR by Starbase. **b** The constructed WT- E2F3 or MUT- E2F3 and miR-7-5p mimic or control miRNA were co-transfected into HK1 and HONE1 cells to detect luciferase activity. **c** Detection of the protein expression of E2F3 in miR-7-5p transfected cells by Western Blot. **p < 0.01

In this experiment, Starbase predicted that E2F3 might be the target of miR-7-5p, and the binding site between 3′-UTR of E2F3 and miR-7-5p was shown in Fig. 4a. The constructed WT-E2F3 or MUT- E2F3 and miR-7-5p mimic or control miRNA were co-transfected into HK1 and HONE1 cells. Double luciferase reporter assays showed that the luciferase activity in HK1 and HONE1 cells co-transfected by WT-E2F3 and miR-7-5p mimic was significantly lower than that in the control group (Fig. 4b, *p *< 0.01). Additionally, the western blot results showed that the protein expression level of E2F3 in HK1 and HONE1 cells transfected with miR-7-5p mimic was significantly lower than that in the control group (Fig. 4c). These results suggested that E2F3 was the target gene of miR-7-5p in NPC cells.

### Effect of circRNA CDR1as on the E2F3expression in NPC tissues and cells

**Fig. 5:**
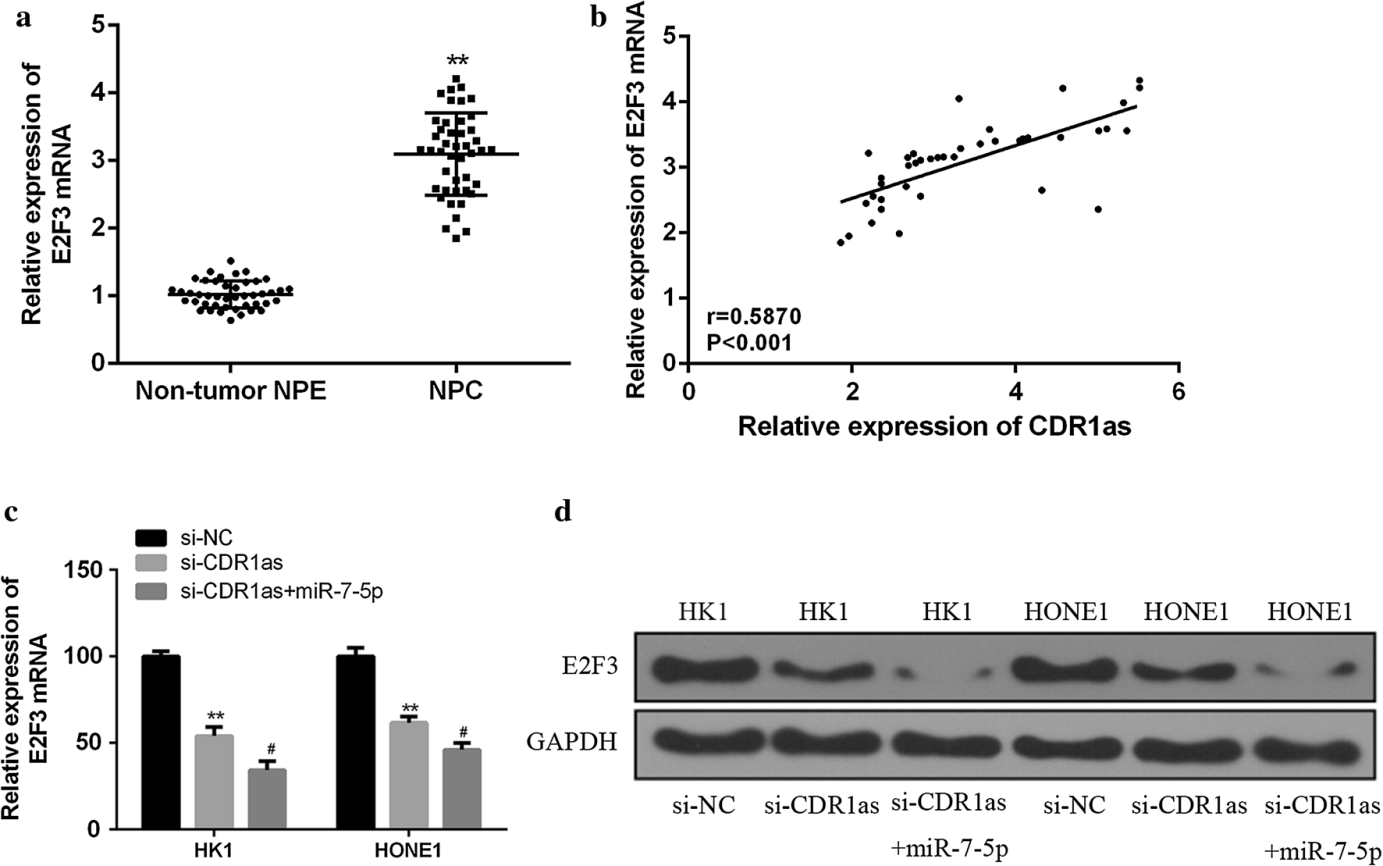
Effect of circRNA CDR1as on the expression of E2F3 in NPC tissues and cells. **a** Determination of the expression levels of E2F3 in NPC tissues and adjacent normal tissues (n = 44). **b** Analyzation of the correlation between E2F3 and circRNA CDR1as expression in NPC tissues by Pearson (n = 30). **c** Determination of the mRNA expression of E2F3 in HK1 and HONE1 cells co-transfected with si-CDR1as or si-CDR1as and miR-7-5p mimic by qRT-PCR. **d** Determination of the protein expression of E2F3 in HK1 and HONE1 cells co-transfected with si-CDR1as or si-CDR1as and miR-7-5p mimic by qRT-PCR. Compared with the si-NC group, **p < 0.01; compared with the si-CDR1as group, ^#^*p* < 0.05

To investigate the effect of circRNA CDR1as on E2F3 expression, si-CDR1as and si-NC were transfected into HK1 and HONE1 cells to determine the expression of E2F3 in NPC tissues and cells. As shown in Fig. 5a, the expression of E2F3 in NPC tissues was significantly higher than that in adjacent normal tissues (*p *< 0.01). Meanwhile, pearson correlation analysis showed a significantly positive correlation between circRNA CDR1as and E2F3 expression (Fig. 5b, *p *< 0.001). In addition, the interference of the CDR1as expression significantly inhibited the mRNA and protein expression levels of E2F3 (*p *< 0.01), and miR-7-5p mimic enhanced this inhibition (Fig. 5c, d, *p *< 0.05). These results indicated that circRNA CDR1 could be competitively bound to miR-7-5p as ceRNA to promote the E2F3expression in NPC.

### Role of miR-7-5p and E2F3 in the growth and glucose metabolism of NPC cells treated with circRNA CDR1as

**Fig. 6:**
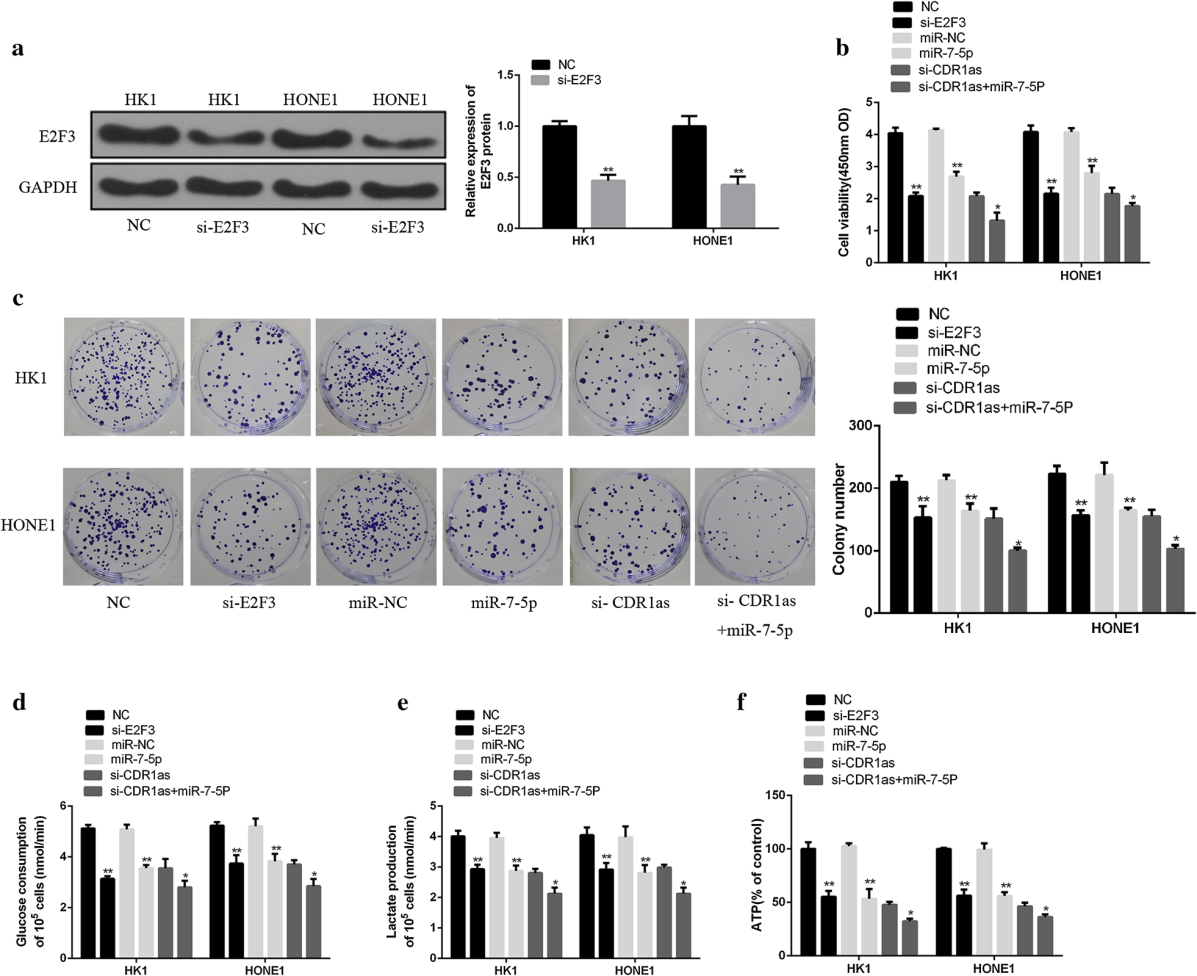
The role of miR-7-5p and E2F3 in promoting the growth and glucose metabolism of NPC cells treated with circRNA CDR1as. **a** Determination of the protein expression of E2F3 after si-E2F3 transfection by Western Blot. **b** Determination of the viability of HK1 and HONE1 cells by CCK8 assay. **c** Measurement of the proliferation of HK1 and HONE1 cells by colony formation assay. **d** Determination of the glucose consumption in HK1 and HONE1 cells. **e** Determination of lactic acid production in HK1 and HONE1 cells. **f** Determination of ATP production in HK1 and HONE1 cells. si-E2F3 vs. NC; miR-7-5P vs. miR-NC; si-CDR1as vs. si-CDR1as + miR-7-5p. *p < 0.05, **p < 0.01

**Fig. 7:**
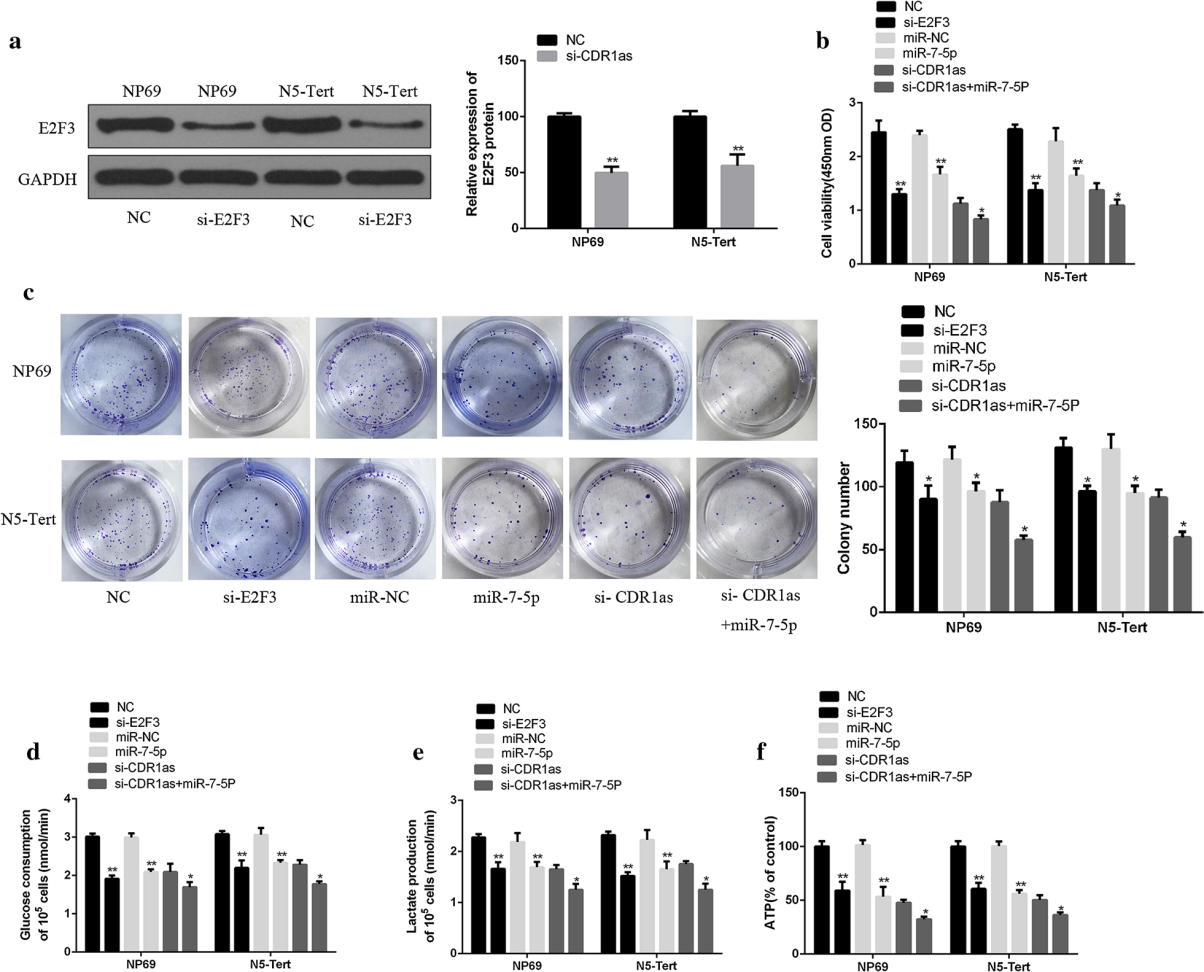
The role of miR-7-5p and E2F3 in promoting the growth and glucose metabolism of normal nasopharyngeal epithelial cells by circRNA CDR1as. **a** Determination of the protein expression of E2F3 after si-E2F3 transfection by Western Blot. **b** Determination of the viability of NP69 or N5-Tert cells by CCK8 assay. **c** Measurement of the proliferation of NP69 or N5-Tert cells by colony formation assay. **d** Determination of the glucose consumption in NP69 or N5-Tert cells. **e** Determination of lactic acid production in NP69 or N5-Tert cells. **f** Determination of ATP production in NP69 or N5-Tert cells. si-E2F3 vs. NC; miR-7-5P vs. miR-NC; si-CDR1as vs. si-CDR1as + miR-7-5p. *p < 0.05, **p < 0.01

Recent studies have shown that E2F3 can promote the growth of tumor cells by accelerating the metabolism of glucose [24]. The si-E2F3, si-CDR1as, and miR-7-5p mimic were transfected into HK1 and HONE1 cells to investigate the role of E2F3 in the growth and glucose metabolism of NPC cells treated with circRNA CDR1as. As shown in Fig. 6a, the protein expression of E2F3 in HK1 and HONE1 cells transfected with si-E2F3 was significantly lower than that in the control group (*p* < 0.01), indicating that si-E2F3 transfection successfully knocked down the E2F3expression. In addition, the interference of E2F3 expression and miR-7-5p overexpression significantly inhibited the growth and glucose metabolism of HK1 and HONE1 cells (Fig. 6b–e, *p* < 0.01). Meanwhile, the growth and glucose metabolism of HK1 and HONE1 cells in the si-CDR1as + miR-7-5p group was significantly inhibited, compared with the si-CDR1as group (Fig. 6b–e, *p* < 0.05). Moreover, similar trends were observed in normal nasopharyngeal epithelial cells, including NP69 and N5-Tert cells (Fig. 7a–e). The above results confirmed that circRNA CDR1as could up-regulate the E2F3expression by binding miR-7-5p, thus promoting the growth and glucose metabolism of NPC cells.

### In vivo effect of circRNA CDR1 on NPC

**Fig. 8:**
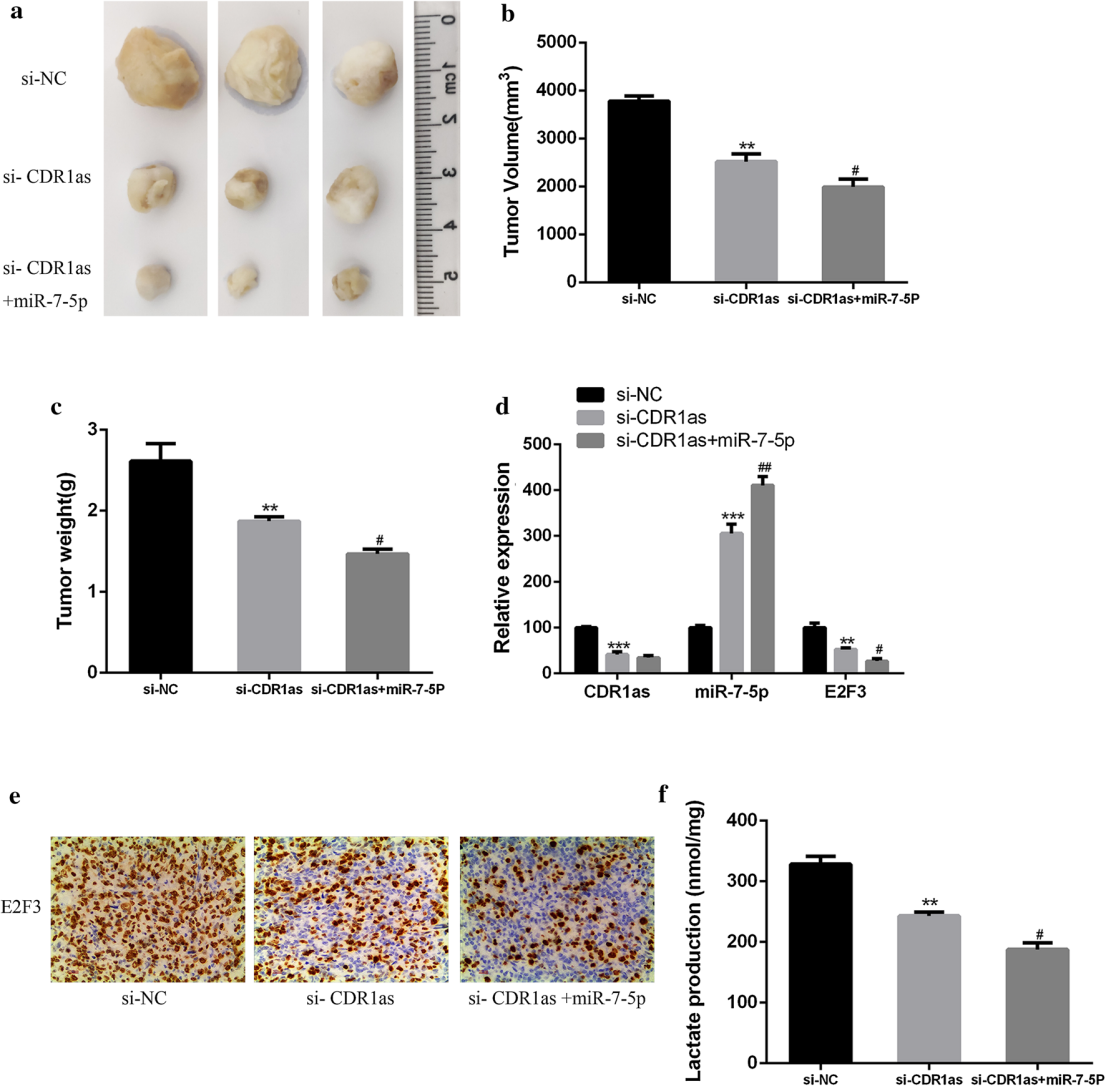
In vivo effect of circRNA CDR1 on NPC. **a**, **b** Measurement of tumor volume. **c** Determination of the tumor weight. **d** Detection of the mRNA expressions of circRNA CDR1as, miR-7-5p and E2F3 by qRT-PCR. **e** Detection of the expression of E2F3 in NPC tissue sections by immunohistochemical staining. **f** Determination of lactic acid level in NPCS tissues. Compared with the si-NC group, **p < 0.01; compared with the si-CDR1as group, ^#^*p *< 0.05

In this study, NPC xenograft model was used to further in vivo investigate the effect of circRNA CDR1as on NPC. As shown in Fig. 8a, the interference of circRNA CDR1as expression significantly inhibited the growth of NPC tumors, compared with the control group. At the same time, the mean volume and weight of tumor in the si- CDR1as group were significantly lower than those in the control group (Fig. 8b, c, *p *< 0.01), and miR-7-5p mimic promoted the tumor inhibition effect of si-CDR1as (*p *< 0.05). In addition, the mRNA expression of miR-7-5p in the si-CDR1as group was significantly up-regulated (Fig. 8d, *p *< 0.01), while the mRNA expression of E2F3 was significantly down-regulated (*p *< 0.01). Furthermore, the immunohistochemical staining showed that the protein expression of E2F3 was also significantly down-regulated in the si-CDR1as group (Fig. 8e). Finally, it was found that the level of lactic acid in the tumor tissues of the si-CDR1as group was significantly reduced (*p *< 0.01), and miR-7-5p mimic promoted this inhibitory effect (Fig. 8f, *p *< 0.05). The above results indicated that circRNA CDR1 could promote the growth of NPC tumors and increase the production of lactic acid in vivo by inhibiting the expression of miR-7-5p and while up-regulating the E2F3 expression.

## Discussion

NPC is head and neck malignant tumor with high incidence, natural local recurrence or metastasis and other pathological characteristics [25]. Although NPC cells are highly sensitive to radiotherapy, NPC patients are suffering from metastasis and poor prognosis [26]. Therefore, valid gene targets is a vital issue in the treatment of NPCs. CircRNA, an endogenous RNA molecule, widely exists in mammalian cells, and plays a role in regulating gene expression [27]. Recent studies have found that circRNA CDR1as is involved in the occurrence and development of liver cancer, bladder cancer, esophageal cancer, ovarian cancer, and other cancers [16, 28]. Xu et al. found that circRNA CDR1as was highly expressed in liver cancer cells and was one of the independent risk factors for the hepatic microvascular invasion in hepatocellular carcinoma [29]. Li et al. reported that circRNA CDR1as was down-regulated in bladder cancer, and over-expressed circRNA CDR1as could play its anti-cancer role by binding to RNA-135a [30]. In this study, the specific molecular mechanism of circRNA CDR1as in NPC tissues and cells was investigated. The results showed that circRNA CDR1as was highly expressed in NPC tissues and cell lines, suggesting that circRNA CDR1 might be related to the promotion of NPC development. In addition, this study also found that the mRNA expression of CDR1as was highest in HK1 and HONE1 cells. Therefore, HK1 and HONE1 cells were selected for subsequent experiments. Tumor cells metabolize glucose into a large amount of lactic acid through aerobic glycolysis, and produce ATP to provide energy for the proliferation and metastasis [31]. Thus, the measurement of glucose metabolism in tumor cells can effectively evaluate the growth status of tumor cells. In this study, si-CDR1as and si-NC were transfected into HK1 and HONE1 cells to investigate the effect of circRNA CDR1as on the growth and glucose metabolism of NPC cells. It was found that the interference of circRNA CDR1as expression significantly inhibited the cell viability and proliferation of HK1 and HONE1 cells. Furthermore, to silence circRNA CDR1as would reduce glucose consumption in HK1 and HONE1 cells and inhibit the accumulation of lactic acid and ATP production. According to the above results, circRNA CDR1as might promote the growth and glucose metabolism of NPC cells.

Studies have shown that circRNAs can act as endogenous RNA (ceRNA) to competitively bind to miRNAs, thus promoting the function of miRNAs target genes [32]. CDR1as is the first circRNA that has been proved to have biological functions in mammalian cells, which can bind to miR-7 and act as inhibitors of miR-7, indirectly increasing the stability of miR-7 target genes [33]. E2F3 is a transcription factor involved in cell proliferation and cycle regulation, and highly expressed in bladder cancer, prostate cancer, and liver cancer [34–36]. To explore the molecular mechanism of CDR1as in NPC, this study used biological information to predict that miR-7-5p was a possible target of CDR1as, and E2F3 was a potential target of miR-7-5p. The results of dual-luciferase reporter gene assay confirmed that miR-7-5p was the target gene of CDR1as in NPC, and E2F3 was the target gene of miR-7-5p in NPC cells.

Meanwhile, it was also found that there was a significantly negative correlation between CDR1as and miR-7-5p expression in NPC tissues, and a significantly positive correlation between CDR1as and E2F3 expression. Moreover, the interference with the CDR1as expression significantly inhibited the E2F3 expression, and miR-7-5p mimics enhanced this inhibitory effect. According to the above results, circRNA CDR1 could be competitively bound to miR-7-5p as ceRNA to promote the E2F3 expression in NPC. Additionally, si-E2F3, si-CDR1as, and miR-7-5p mimic were transfected into HK1 and HONE1 cells to investigate whether miR-7-5p and E2F3 were related to circRNA CDR1as-mediated NPC cell growth and glucose metabolism. In normal nasopharyngeal epithelial cells, it revealed that miR-7-5p and E2F3 promoted the growth and glucose metabolism, observed by Western Blot, CCK8, colony formation assay, and the determinations of glucose consumption, lactic acid, and ATP production in NP69 or N5-Tert cells. The results showed that circRNA CDR1as could up-regulate the E2F3 expression by binding miR-7-5p, thus promoting the growth and glucose metabolism of NPC cells.

Animal model of the human tumor is an indispensable means to study human tumor. The establishment of the animal model of NPC transplantation tumor provides an ideal experimental material and method for the study of NPC [37]. To further investigate the in vivo effect of circRNA CDR1as on NPC, we established an animal model of NPC xenograft tumor and treated mice with si-CDR1as and miR-7-5p mimic. The results showed that the average tumor size and weight in the si-CDR1as group were significantly lower than those in the control group, suggesting that circRNA CDR1as interference could dramatically inhibit the growth of NPC tumors, and miR-7-5p mimics enhanced this inhibitory effect. In addition, the mRNA expression of miR-7-5p in the si-CDR1as group was significantly up-regulated, while the mRNA nucleoprotein expression of E2F3 was significantly down-regulated. We also found that the lactate level in tumor tissues of the si-CDR1as group was reduced considerably, and miR-7-5p mimic enhanced this inhibitory effect. The above results confirmed that circRNA CDR1 could in vivo promote the growth and glucose metabolism of NPC tumors by inhibiting the miR-7-5p expression and up-regulating the E2F3 expression.

Accelerated glycometabolism is one of the major causes of accelerated proliferation of NPC cells. Our study revealed that CDR1as promoted the proliferation of NPC cells, and the acceleration of glycometabolism could be observed simultaneously. However, glycometabolism is not the only factor for CDR1as-induced cell proliferation. In the future, we will conduct more experiments to elucidate all other possible causes of accelerated proliferation of NPC cells.

## Conclusion

In conclusion, the present study evaluated the molecular mechanism of circRNA CDR1as in the growth and glucose metabolism of NPC tumors. The results confirmed that circRNA CDR1as could up-regulate the expression of E2F3 by binding miR-7-5p, thus promoting the occurrence and development of NPC. It provided a potential target for the treatment of NPC patients.

## Data Availability

All data generated or analyzed during this study are included in this published article.

## Abbreviations

circRNA: circular RNA
NPC: nasopharyngeal carcinoma
CCK8: cell counting kit-8
miRNA: micro RNA
qRT-PCR: quantitative real-time polymerase chain reaction
ceRNA: competitive endogenous RNAs
FBS: fetal bovine serum
CDR1as: cerebellar degeneration associated protein 1 antisense transcript
PVDF: polyvinylidene difluoride
NAT: natural antisense transcript
E2F3: E2F transcription factor 3

## References

[1] Ahmed HG, Suliman RSAG, Aziz MSAE, Alshammari FD (2015). Molecular screening for Epstein Barr virus (EBV) among Sudanese patients with nasopharyngeal carcinoma (NPC). Infect Agents Cancer.

[2] Li LX, Tian W, Wang WY, Liu KL, Wang JL, Jin HK, Cai JH, Wang JJ (2015). NKG2C copy number variations in five distinct populations in mainland China and susceptibility to nasopharyngeal carcinoma (NPC). Hum Immunol.

[3] Lan Z, Fong AHW, Na L, Cho WCS (2018). Molecular subtyping of nasopharyngeal carcinoma (NPC) and a microRNA-based prognostic model for distant metastasis. J Biomed Sci.

[4] Qiu Y, Guo Z, Han L, Yang Y, Li J, Liu S, Lv X (2017). Network-level dysconnectivity in patients with nasopharyngeal carcinoma (NPC) early post-radiotherapy: longitudinal resting state fMRI study. Brain Imaging Behav.

[5] Kam MKM, Wong FCS, Kwong DLW, Sze HCK, Lee AWM (2014). Current controversies in radiotherapy for nasopharyngeal carcinoma (NPC). Oral Oncol.

[6] Ainscow EK, Brand MD (2010). Top-down control analysis of ATP turnover, glycolysis and oxidative phosphorylation in rat hepatocytes. FEBS J.

[7] Tourmente M, Villarmoya P, Rial E, Roldan ER (2015). Differences in ATP generation via glycolysis and oxidative phosphorylation and relationships with sperm motility in mouse species. J Biol Chem.

[8] Mathew J, Loranger A, Gilbert S, Faure R, Marceau N (2013). Keratin 8/18 regulation of glucose metabolism in normal versus cancerous hepatic cells through differential modulation of hexokinase status and insulin signaling. Exp Cell Res.

[9] Zhang S, Zeng X, Ding T, Guo L, Li Y, Ou S, Yuan H (2018). Microarray profile of circular RNAs identifies hsa_circ_0014130 as a new circular RNA biomarker in non-small cell lung cancer. Sci Rep.

[10] Huang Z, Su R, Qing C, Peng Y, Luo Q, Li J (2010). Plasma circular RNAs hsa_circ_0001953 and hsa_circ_0009024 as diagnostic biomarkers for active tuberculosis. Front Microbiol.

[11] Zheng Q, Bao C, Guo W, Li S, Chen J, Chen B, Luo Y, Lyu D, Li Y, Shi G (2016). Circular RNA profiling reveals an abundant circHIPK3 that regulates cell growth by sponging multiple miRNAs. Nat Commun.

[12] Xu H, Guo S, Li W, Yu P (2015). The circular RNA Cdr1as, via miR-7 and its targets, regulates insulin transcription and secretion in islet cells. Sci Rep.

[13] Zhu W, Wang Y, Zhang D, Yu X, Leng X (2018). MiR-7-5p functions as a tumor suppressor by targeting SOX18 in pancreatic ductal adenocarcinoma. Biochem Biophys Res Commun.

[14] Luo H, Liang H, Chen Y, Chen S, Xu Y, Xu L, Liu J, Zhou K, Peng J, Guo G (2018). miR-7-5p overexpression suppresses cell proliferation and promotes apoptosis through inhibiting the ability of DNA damage repair of PARP-1 and BRCA1 in TK6 cells exposed to hydroquinone. Chem Biol Interact.

[15] Liu Z, Liu Y, Li L, Xu Z, Bi B, Wang Y, Li JY (2014). MiR-7-5p is frequently downregulated in glioblastoma microvasculature and inhibits vascular endothelial cell proliferation by targeting RAF1. Tumor Biol.

[16] Yu L, Gong X, Sun L (2016). The circular RNA Cdr1as act as an oncogene in hepatocellular carcinoma through targeting miR-7 expression. PLoS ONE.

[17] Li X, Zheng Y, Zheng Y, Huang Y, Zhang Y, Jia L, Li W (2018). Circular RNA CDR1as regulates osteoblastic differentiation of periodontal ligament stem cells via the miR-7/GDF5/SMAD and p38 MAPK signaling pathway. Stem Cell Res Ther.

[18] Xue Y, Xiong Q, Wu Y, Li S, Ge F (2017). Quantitative proteomics reveals the regulatory networks of circular RNA CDR1as in hepatocellular carcinoma cells. J Proteome Res.

[19] Sang M, Meng L, Sang Y, Liu S, Ding P, Ju Y, Liu F, Gu L, Lian Y, Li J (2018). Circular RNA ciRS-7 accelerates ESCC progression through acting as a miR-876-5p sponge to enhance MAGE-A family expression. Cancer Lett.

[20] Guo Y, Qi Y, Guo A, Du C, Zhang R, Chu X (2017). miR-564 is downregulated in gastric carcinoma and targets E2F3. Oncol Lett.

[21] Lei L, Qiu M, Tan G, Liang Z, Yue Q, Chen L, Chen H, Liu J (2014). miR-200c inhibits invasion, migration and proliferation of bladder cancer cells through down-regulation of BMI-1 and E2F3. J Transl Med.

[22] Kang BJ, Wang Y, Zhang L, Li SW (2017). Basic fibroblast growth factor improved angiogenesis of vitrified human ovarian tissues after in vitro culture and xenotransplantation. Cryo Lett.

[23] Chan JJ, Tay Y (2018). Noncoding RNA:RNA regulatory networks in cancer. Int J Mol Sci.

[24] Luan W, Zhou Z, Ni X, Xia Y, Wang J, Yan Y, Xu B (2018). Long non-coding RNA H19 promotes glucose metabolism and cell growth in malignant melanoma via miR-106a-5p/E2F3 axis. J Cancer Res Clin Oncol.

[25] Hou Y, Zhu Q, Li Z, Peng Y, Yu X, Yuan B, Liu Y, Liu Y, Yin L, Peng Y (2017). The FOXM1–ABCC5 axis contributes to paclitaxel resistance in nasopharyngeal carcinoma cells. Cell Death Dis.

[26] Kang M, Zhou P, Long J, Li G, Yan H, Feng G, Liu M, Zhu J, Wang R (2017). A new staging system for nasopharyngeal carcinoma based on intensity-modulated radiation therapy (IMRT). Oncotarget.

[27] Legnini I, Di TG, Rossi F, Morlando M, Briganti F, Sthandier O, Fatica A, Santini T, Andronache A, Wade M (2017). Circ-ZNF609 is a circular RNA that can be translated and functions in myogenesis. Mol Cell.

[28] Liang HF, Zhang XZ, Liu BG, Jia GT, Li WL (2017). Circular RNA circ-ABCB10 promotes breast cancer proliferation and progression through sponging miR-1271. Am J Cancer Res.

[29] Xu L, Zhang M, Zheng X, Yi P, Lan C, Xu M (2017). The circular RNA ciRS-7 (Cdr1as) acts as a risk factor of hepatic microvascular invasion in hepatocellular carcinoma. J Cancer Res Clin Oncol.

[30] Li P, Yang X, Yuan W, Yang C, Zhang X, Han J, Wang J, Deng X, Yang H, Li P (2018). CircRNA-Cdr1as exerts anti-oncogenic functions in bladder cancer by sponging microRNA-135a. Cell Physiol Biochem Int J Exp Cell Physiol Biochem Pharmacol.

[31] Xiao Q, Wei Z, Li Y, Zhou X, Chen J, Wang T, Shao G, Zhang M, Zhang Z (2018). miR-186 functions as a tumor suppressor in osteosarcoma cells by suppressing the malignant phenotype and aerobic glycolysis. Oncol Rep.

[32] Xiong DD, Dang YW, Lin P, Wen DY, He RQ, Luo DZ, Feng ZB, Chen G (2018). A circRNA–miRNA–mRNA network identification for exploring underlying pathogenesis and therapy strategy of hepatocellular carcinoma. J Transl Med.

[33] Hansen TB, Kjems J, Damgaard CK (2013). Circular RNA and miR-7 in cancer. Can Res.

[34] Huang L, Luo J, Cai Q, Pan Q, Zeng H, Guo Z, Dong W, Huang J, Lin T (2011). MicroRNA-125b suppresses the development of bladder cancer by targeting E2F3. Int J Cancer.

[35] Tao T, Liu D, Liu C, Xu B, Chen S, Yin Y, Ang L, Huang Y, Zhang X, Chen M (2014). Autoregulatory feedback loop of EZH2/miR-200c/E2F3 as a driving force for prostate cancer development. Biochim Biophys Acta.

[36] Li W, Ni GX, Zhang P, Zhang ZX, Li W, Wu Q (2010). Characterization of E2F3a function in HepG2 liver cancer cells. J Cell Biochem.

[37] Sides MD, Sosulski ML, Luo F, Lin Z, Flemington EK, Lasky JA (2013). Co-treatment with arsenic trioxide and ganciclovir reduces tumor volume in a murine xenograft model of nasopharyngeal carcinoma. Virol J.

